# Warming in the Arctic Captured by productivity variability at an Arctic Fjord over the past two centuries

**DOI:** 10.1371/journal.pone.0201456

**Published:** 2018-08-15

**Authors:** Vikash Kumar, Manish Tiwari, R. Rengarajan

**Affiliations:** 1 National Centre for Antarctic & Ocean Research, Vasco-da-Gama, Goa, India; 2 Physical Research Laboratory, Navrangpura, Ahmedabad, India; Centro de Investigacion Cientifica y de Educacion Superior de Ensenada Division de Fisica Aplicada, MEXICO

## Abstract

Arctic fjords feature among some of the most climate-sensitive regions on the planet. The site of this study–Kongsfjorden–is one such fjord in which sedimentation and sediment geochemistry reflect climate-mediated changes in glacial melt and marine primary productivity. Here, we have shown that the fjord is particularly sensitive to the changing melt dynamics of the surrounding glaciers which are a direct consequence of warming/cooling in the region and is reflected in the productivity at the fjord. Warming increases meltwater influx into the fjord leading to enhanced turbidity which results in lower productivity. A multi-proxy study (sedimentary organic matter content, carbon and nitrogen isotope ratios, and microfossil abundance) using a 21 cm long sediment core from the Kongsfjorden helped us reconstruct warming driven melt-dynamics history for the past two centuries. Proxy data show a general decreasing trend in productivity along with a few excursions over the last two centuries. Warming driven glacial-melt dynamics appears to be the dominant control on productivity throughout the span of the core.

## Introduction

An amplified response to ongoing climate change is widely reported from the Arctic region [1,2]. Some of the most visible changes in the region, as seen through instrumental records and beyond are the rapidly rising temperature [3] and shrinking sea ice cover [4]. The region-wide warming over the last century is estimated to be twice that of the global average [5]. Cascading effects of these changes in the Arctic region are projected to significantly alter the state and the balance of the climate system into the lower latitudes where most of the civilization resides. These include large-scale changes in ocean circulations, air-sea interaction and enhanced loading of greenhouse gases into the atmosphere [6–9]. As the instrumental records from the Arctic region are short and sparse [10], there is a need for additional paleoclimatic studies. Although various ice core studies from the region have been remarkably successful in extending its past climate history, attention to other paleoclimate proxies from the region are still inadequate. While ice-core proxies offer good regional and global scale reconstruction, various geochemical sedimentary proxies such as those related to primary production (hereafter referred to as ‘productivity’) can potentially help link local environmental responses to regional scale climate changes.

**Fig 1:**
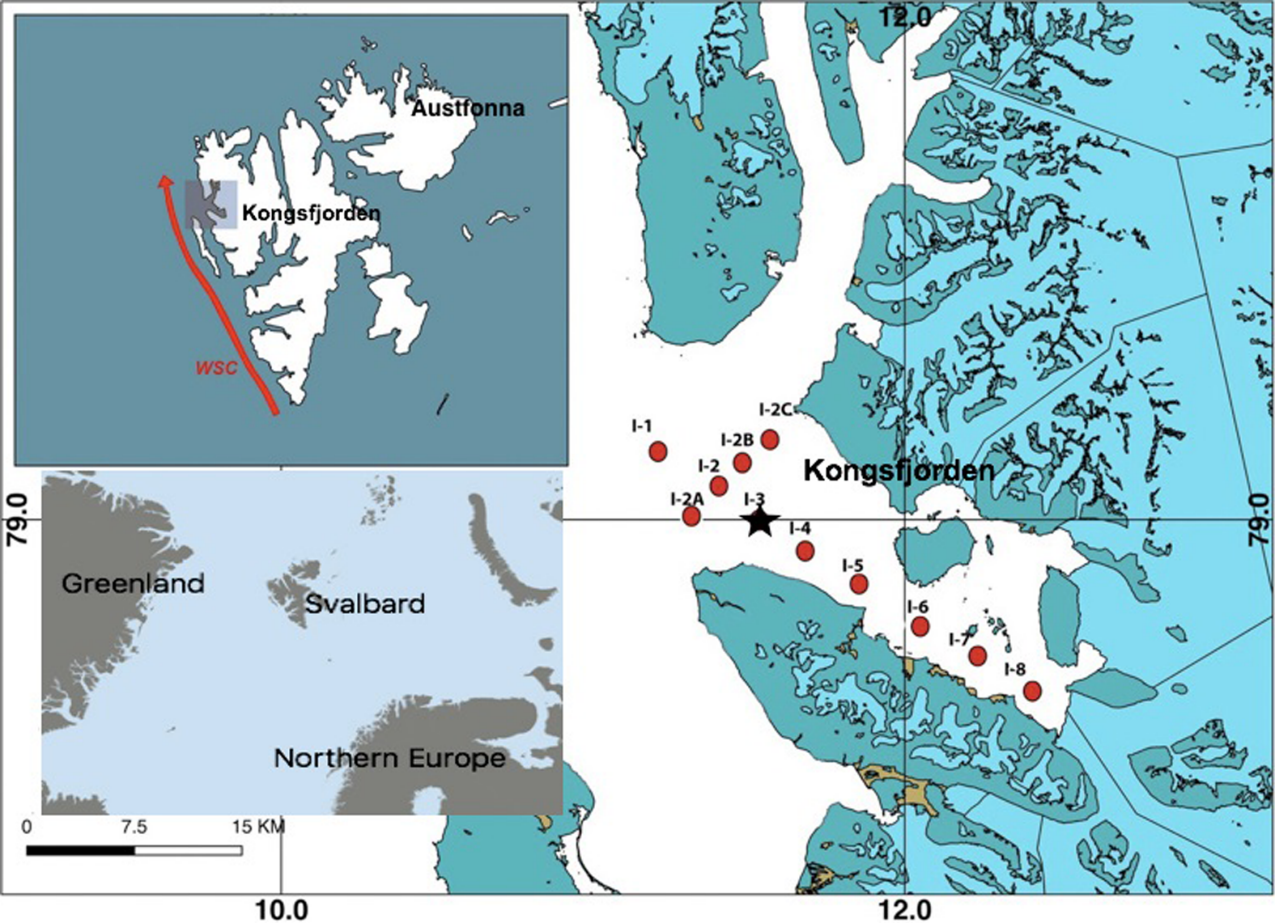
Map of Kongsfjorden in Svalbard showing sampling locations. Location where sediment core was collected is marked with a star.

Sedimentary geochemical proxies offer a multi-proxy reconstruction of past productivity changes.[11]. These are primarily bulk sediment parameters such as organic matter concentration and its carbon and nitrogen isotopic composition and can be readily analyzed in surface and core sediments (detailed introduction of geochemical proxies provided under the section “Sedimentary geochemical proxies of productivity and its spatial variability at Kongsfjorden”). However, linking these productivity proxies at a specific location to large-scale warming and glacial melting require a detailed investigation of the site-specific dynamics, preferably at a place where melting plays an important role in marine productivity. Glaciers have been known to impact productivity by influencing the inorganic supply and physical conditions within fjord systems [12–15]. The location of this study is a glacier-fed Arctic fjord—Kongsfjorden–in Svalbard where glacial melting is a dominant factor (Fig 1). Here our primary objective is to reconstruct productivity changes in Kongsfjorden over the last 200 years and assess whether productivity changes are linked to changes in temperature derived from instrumental and ice core records.

### Study-area

Kongsfjorden is located in the western part of Spitsbergen Island of the Svalbard archipelago. Situated between 78° 40’-77° 30’ N and 11° 3’-13° 6’ E, it is oriented from southeast to northwest along a 20 km stretch. Its width varies between 4 km towards the head and 10 km towards the mouth. The shallow inner part consists of less than 100 m of water depth whereas the outer part is deeper at ~ 300 m. With an overall 80% glaciated catchment, two active tidewater-glaciers towards the head (Kongsvegen and Kronebreen) and three towards the northern coast (Blomstandbreen, Conwaybreen, and Kongsbreen) feed into the fjord. No tidewater-glacier is present near the southern coast. The glacial-marine contrast at Kongsfjorden gives rise to a strong hydrological, sedimentological and biological gradient along the axis of the fjord [16]. With a high glacial sedimentation, the site offers the the potential for developing a high resolution climate reconstruction.

### Linking glacial melting and productivity at Kongsfjorden

#### Glacial runoff and sedimentation

Svalbard glaciers represent 4% of the world’s land-ice [17], equivalent to ~ 17 mm of sea-level [18]. Glacial mass balance is a function of air-temperature, radiation and precipitation. The melt-water produced by warming in the ice-covered region, apart from contributing directly to the loss of mass, also reduce basal friction and accelerates the process of calving and frontal ablation in tidewater glaciers. Melting of ice caps and glaciers in Svalbard has contributed to 0.01 mm y^-1^ of global sea level rise over the last three decades [19]. The major contribution of freshwater at Kongsfjorden comes from glacial discharge with limited supply from precipitation [20,21]. Together the glacier complex at Kongsfjorden drains close to 1 km^3^ of freshwater into the fjord annually with significant inter-annual variations [21]. The inter-annual variabilities in meltwater run-off are driven by summer mass balance, suggesting the importance of temperature in glacial ablation and calving. Glacial runoff is a major source of sediment supply to the basin where a large part is deposited in the inner part of the fjord. In the inner part, melt-water enters the fjord flowing at >50 cm s^-1^ with close to 500 mg l^-1^ of sediment in suspension [22]. Sediment accumulation rate at the glacial front in the inner part of the fjord (20,000 g m^-2^ y^-1^) is an order of magnitude higher than those at the central fjord and two orders of magnitude higher than those in the outer part [21]. Such a steep gradient in sediment load give rise to highly turbid water in the inner part with progressively clear water towards the open ocean. Temperature in Svalbard has increased by 0.27°C decade^-1^ since 1912 [23] and model projections show that annual-mean temperatures in this region could rise between 7°C and 11°C by the end of this century (IPCC AR5) which may lead to a significant increase in glacial runoff and sediment delivery in the fjord.

#### Physical conditions

Kongsfjorden is influenced by both Atlantic and Arctic water. Western Spitsbergen is flanked by a northerly warm West Spitsbergen Current (WSC), with Atlantic water mass (AW) constituting the top 600 m and a cold coastal current with Arctic water (ArW) traverses the shelf in the outer Fjord region [21].Cross-shelf exchange of these water masses with the inner less saline water is possible due to associated frontal instabilities [24]. North Atlantic Oscillation influences WSC strength [25] and therefore can impact marine ecosystem at Kongsfjorden by increasing the salinity and temperature by advection of Atlantic water. Water mass in the fjord can be vertically described as consisting of three distinct layers of (1) winter-homogenized bottom water, (2) intermediate advected water and (3) local surface water [26]. Winds are largely governed by orographic steering of the large-scale geostrophic field and katabatic flow (detailed discussion presented in [21]).

#### Patterns of primary production

A large proportion of the annual inventory of primary production by pelagic and benthic communities in high Arctic fjords is limited to the spring bloom period [27]. Insufficient light during polar winter eliminates any production and over-grazing during summer slows it down. Various estimates put the annual production at Kongsfjorden between 4 and 180 g C m^-2^ y^-1^, indicating large temporal variability [28]. Both model-based studies [29] and observations have long suggested that sediment loading in turbid estuarine environments strongly impacts primary production by limiting light availability [30], which usually give rise to a steep axial gradient in the depth of the euphotic zone. This is particularly true for the glacial fjord Kongsfjorden where, on an average, a five-fold gradient in the depth of the euphotic zone between the inner and the outer fjord is observed [31,32]. During peak summertime, when glacial run-off is at its maximum, the euphotic zone in the inner part of the fjord can further decrease to as little as 0.3 m compared to ~30 m in the outer part i.e., a hundred-fold decrease [33]. As a result, various estimates of the spatial and temporal distribution of phytoplankton biomass at Kongsfjorden invariably shows distinctly higher concentration in the outer part in comparison to the inner fjord (Hop et al., 2002 and references therein). For example, one study [15] during July 2002 observed the depth-integrated phytoplankton biomass in the outer fjord as 2,770 mg C m^−2^ while it was only 254 mg C m^−2^ towards the inner reaches, showing a steep gradient as also seen in the depth of the euphotic zone. Thus, significant perturbations in glacial discharge over time can be a dominant control on phytoplankton biomass at Kongsfjorden. The presence of a large number of communities representing Atlantic biogeography suggests a strong Atlantic influence on the phototrophic assemblage, however, the role of changing Atlantic water advection on gross production at Kongsfjorden is largely unknown and may possibly be not very significant in comparison to those driven by glacial changes.

#### Sedimentary geochemical proxies of productivity and its spatial variability at Kongsfjorden

Organic carbon and nitrogen content in sediments along with their isotopic composition (δ^13^C and δ^15^N) and microfossil abundance serve as excellent indicators of past productivity changes. Both land-transported refractory material and in situ marine biomass contribute to the organic matter content of the deposited sediment. δ^13^C of the bulk sediment is characteristic of the mixing ratio between the land and the marine-derived organic matter as the two endmembers have widely distinct δ^13^C values. [34]. Thus, bulk δ^13^C can track changes in productivity due to changing marine primary production if terrestrial component roughly remains static. Preferential uptake of ^14^N during nitrate assimilation by autotrophs results in ^15^N enrichment of the ambient inorganic nitrogen pool. Consequently, δ^15^N and ambient nitrate concentration show an inverse relation with each other [35]. If the supply of nitrate is in a steady-state, δ^15^N of the sedimentary record will reflect changes in nitrate consumption and thereby primary production. Apart from the bulk organic geochemical characteristics of sediment, its micropaleontology may also offer valuable insights about changing conditions. Foraminifera assemblage at a particular location is characteristic of the prevailing physical and biological conditions [36–38]. Foraminifera microfossil abundance based on the distribution of dominant foraminifera species in sediment cores can be used as indicators of prevailing conditions [37]. Together, these geochemical and microfossil proxies in sediment core can provide valuable information about past environmental changes, especially, those related to the overlying productivity.

**Fig 2:**
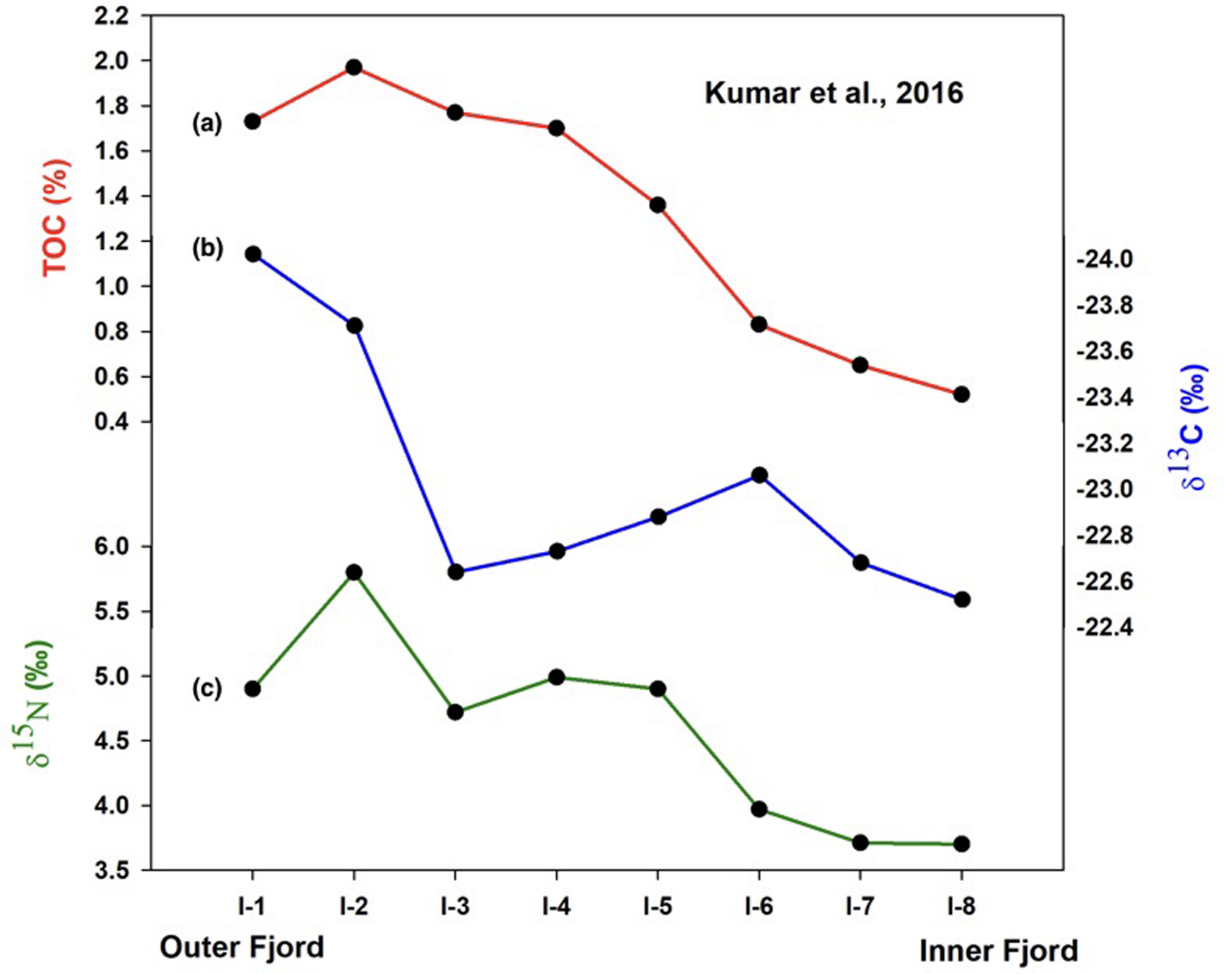
**Spatial variability of (a) TOC, (b) δ^13^C and (c) δ^15^N along the fjord axis at Kongsfjorden (surface sediment data taken from [39])**.

Geochemical proxies in surface sediment [39] along a transect from the glacier front to open mouth at Kongsfjorden captures the steep gradient in productivity shown by various modern observations and estimates of production along the fjord axis. Organic matter concentration as shown by TOC in surface sediments in the inner fjord was found to be quarter of its value in the outer region (Fig 2A). Anomalous end-member marine and terrestrial δ^13^C values with heavier terrestrial carbon isotope ratios and lighter marine carbon isotope ratio were reported based on surface sediments. While the low δ^13^C of marine synthesized carbon may have been due to high pCO_2_ in cold waters, enriched δ^13^C values with elevated C/N ratios for the terrestrial endmember may possibly be due to the presence of ancient marine shale in the eroded material. Thus, low δ^13^C (−24‰) in the outer part compared to the inner part of the fjord (−22.5‰) (Fig 2B) showed a high marine contribution to sedimentary organic matter in the outer region and very low contribution in the inner part along with a trend between the two locations. Similarly, δ^15^N of surface sediments was found to be considerably lower in the inner part (Fig 2C), seemingly due to low nitrate uptake on account of lower productivity in the inner region. Thus, the surface sediment data shows the effectiveness of geochemical proxies in recording glacially driven productivity changes at Kongsfjorden.

## Methods

India possess MoU/treaty with Norwegian authorities to carry out research activities in the Svalbard so no specific permission is required. Field studies did not involve any endangered or protected species.

A sediment core of length 21 cm from the mid-fjord region and eight surface sediment grabs distributed longitudinally were collected during August 2014 from various locations with different water depths at Kongsfjorden (Table 1). Surface sediments were collected using Grab Sampler and sediment core was obtained using a Haps Corer onboard workboat “MS Teisten”. The samples have been named as per their station number (red dots in Fig 1). The sediment core was collected at station I-3 (shown with a star in Fig 1) and was sub-sampled at 1 cm interval onboard. All the samples were freeze-dried before being transported for further processing and analysis. A portion of the freeze-dried samples was finely ground for homogenization and further sub-sampled into two batches—(i) 2N HCl treated batch (ii) untreated batch. By adding approximately 20 ml of 2N HCl solution to 500–1000 mg of finely ground sediment, carbonate was removed. The mixture was swirled and allowed to stand overnight. The samples were then washed with double-distilled water and approximately 5 mg of treated sample was used for TOC and δ^13^C analysis. For nitrogen and its isotopic composition (δ^15^N), approximately 50 mg of untreated sediment was used as acid treatment is known to cause bias in δ^15^N values [40]. The analysis was carried out on an Isotope Ratio Mass Spectrometer coupled to an Elemental Analyzer at the Marine Stable Isotope Lab (MASTIL) at National Centre for Antarctic & Ocean Research, Goa, India. The precision of the organic carbon and nitrogen concentration determinations were 0.31% (1σ standard deviation) and 0.24% (1σ), respectively, based on a Sulfanilamide standard while that of carbon and nitrogen isotopic composition was 0.05 ‰ (1σ) and 0.12 ‰ (1σ) respectively (IAEA-CH-3 and IAEA-N-1 standards). For the foraminifera microfossil study, approximately 20 g of dried sediment was used. 20 ml of 6% hydrogen peroxide solution was added to oxidize any organic matter present in the samples while 10 ml of 10% sodium hexametaphosphate solution was added as a dispersing agent to avoid agglutination of the suspended matter. The wet mixture was washed through a 64 μm sieve and then through a 150 μm sieve on the oven dried fraction. Approximately 300 tests from each sample were identified and analyzed for species identification using a stereo microscope.

**Table 1:** Sampling details.

Sample	Depth	Lattitude (°N)	Longitude (°E)
I-1	250 m	79.0354	11.2836
I-2	254 m	78.9931	11.5547
I-3	294 m	78.9755	11.6915
I-4	180 m	78.9587	11.8224
I-5	302 m	78.9408	11.9576
I-6	145 m	78.9228	12.0937
I-7	74 m	78.9931	12.3000
I-8	45 m	78.8951	12.3201

Sediment core was collected at sampling station I-3

^210^Pb analysis of sediment samples was carried out to obtain mean sedimentation rate at the coring location. For this, the top 12 consecutive sediment samples (0–12 cm core depth) were used. About 4 g of dried homogenized sediment was packed and sealed in a plastic vial and was assayed for ^210^Pb and ^226^Ra after three weeks from sealing the vial to allow ^222^Rn and its daughters to grow into equilibrium with ^226^Ra. The radioactivity analysis was carried out using a high purity Ge well detector coupled with Digital Spectrum Analyzer (GCW4023, Canberra, USA) with a well depth of 35 mm and diameter of 20.5 mm. The resolution of the detector is 1.4 keV (FWHW) at 1.22 MeV. The gamma peak for ^210^Pb is at 46 keV and the gamma peaks for ^226^Ra are at 295, 351 and 609 keV.

## Results

### Chronology

**Fig 3:**
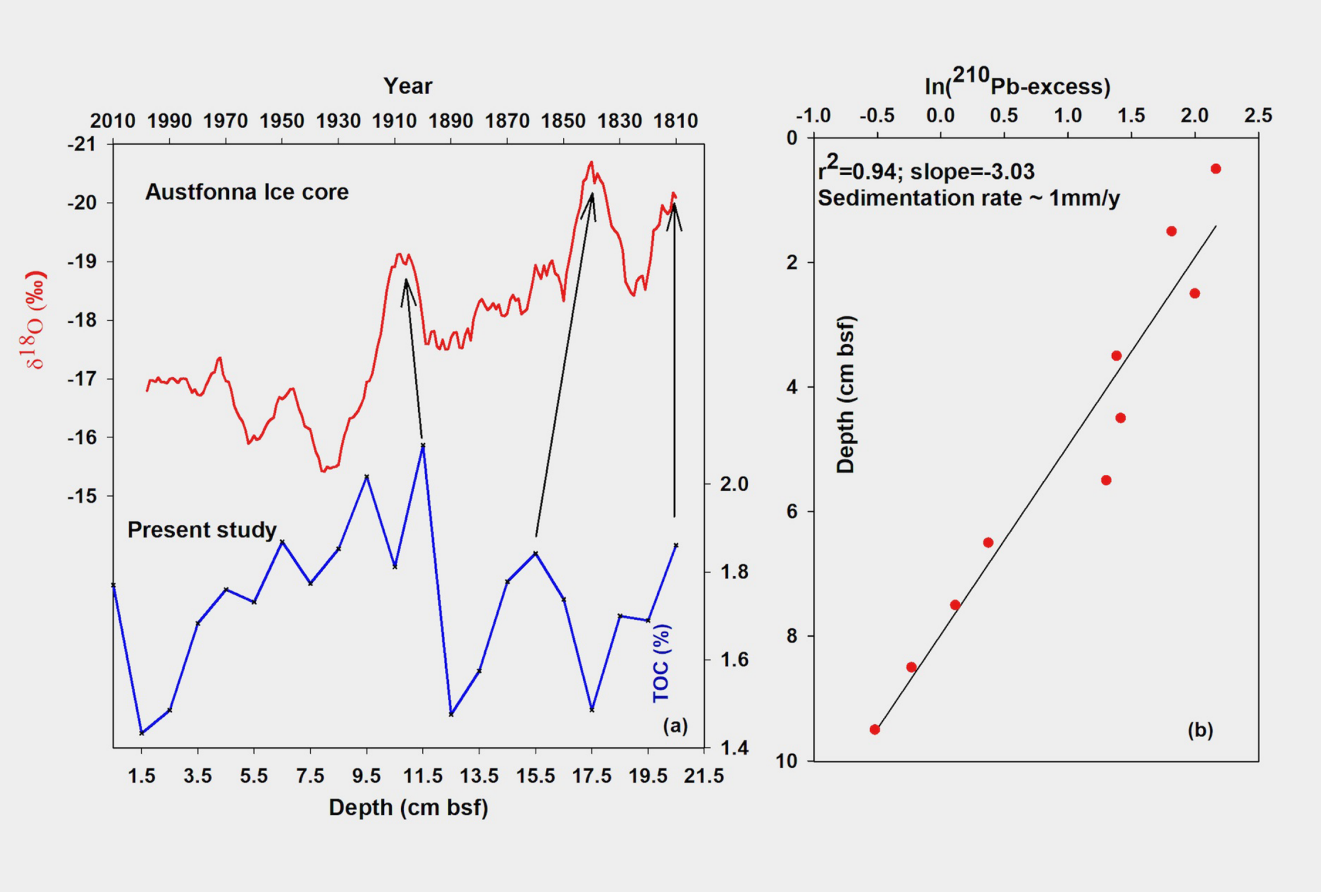
**Chronology of the sediment core established using (a) tie-points in proxy records (10 year averaged δ^18^O record of Austfonna ice-core, and down-core TOC variability as reported in the present study) and (b) sediment accumulation rate calculated using vertical profile of excess ^210^Pb activity**.

The chronology of the 21 cm long sediment core was established using the down core variability of unsupported ^210^Pb activity overlaid with adjustments using peak-matching of geochemical data with a well-dated nearby ice-core record [41] from Svalbard (Fig 3). The radioactivity analysis detected significant levels of unsupported ^210^Pb in the top 11 cm, and the total ^210^Pb activities were in equilibrium with the ^226^Ra activity below this depth (S1 Fig). The choice of lowest total ^210^Pb at 12 cm depth, as a measure of supported ^210^Pb, is justified by ^226^Ra activity at this level (S1 Fig), considered to be in secular equilibrium with the supported fraction of ^210^Pb. Slightly higher ^226^Ra activity (2 dpm/g) compared to total ^210^Pb activity at this level (1.85 dpm/g) may be attributed to minor disequilibrium between the two [42].The excess ^210^Pb activity showed a simple exponential relationship with depth, indicating that the chronology of this profile can be estimated using the Constant Initial Concentration (CIC) model. The mean sedimentation rate of ~1 mm/y was calculated from the slope of the profile using a least-squares fit (r^2^ = 0.94) (Fig 3B). Synchronous peaks among multiple proxies in the geochemical record were used as tie-points with respective peaks in the smoothed ice-core record (smoothed using a 10-year running mean). (Fig 3A). Instrumental observations are not available for the 19^th^ century, thus, a nearby ice-core record was chosen for this purpose. Applying a sedimentation rate of 1 mm y^-1^ above the 11.5 cm horizon in the core yielded the core top date as AD 2010 while the core bottom date came out as AD 1810 at 21 cm depth. Thus, the 21cm sedimentary record corresponds to the last two centuries (AD 1810 –AD 2010).

### Sedimentary organic matter and its isotopic composition in core sediments

**Fig 4:**
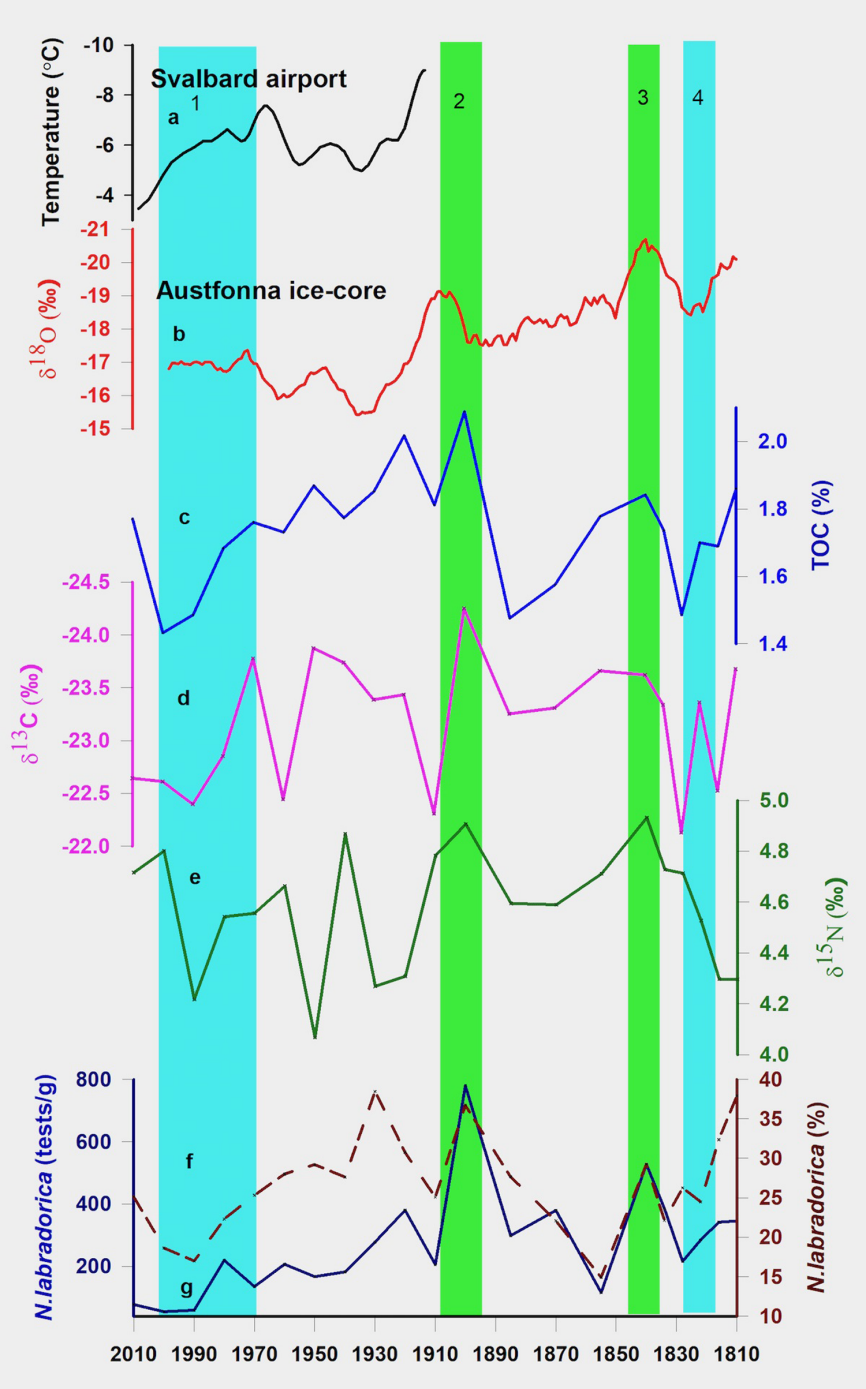
**(a) Svalbard airport temperature record (b) δ^18^O of Austfonna ice-core record from Svalbard and down-core variability of (c) TOC, (d) δ^13^C, (e) δ^15^N, and (f,g) microfossil abundance of *Nonionellena labradorica*, in the present study**.

The organic carbon concentration in the sediment core varied between 1.3% and 2.1% during the period AD 1810 and AD 2010 (Fig 4C). Nitrogen content varied between 0.19% and 0.13% during this period. Peak concentrations were observed around AD 1840 and AD 1900. After a short-lived abrupt decline in TOC during the early 19th century, rest of the century witnessed a gradual decline in organic matter concentration post the AD 1840 peak. Overall, TOC during the late 19^th^ century was significantly lower than those during the early 19^th^ century. TOC during the 20^th^ century shows a more prominent declining trend post the AD 1900 peak. The decline appears particularly accelerated after 1970, except for the top-most section of the core where considerably high TOC was found.

δ^13^C of organic matter in the sediment core varied between -22.25 ‰ and -24.25 ‰ with a mean of -23.17 ‰ during the last two centuries (Fig 4D). Trends are similar to those shown by TOC during this period with prominent synchronous peaks at AD 1840 and AD 1900. A moderately strong negative correlation (r = -0.62) between TOC and δ^13^C (S2 Fig) was found for the entire period (r = -0.66 during the 20^th^ century and r = -0.76 during the 19^th^ century). After an abrupt excursion during the early 20^th^ century, δ^13^C showed a slight increase during the remainder of the century post the AD 1840 peak. δ^13^C was in general higher during the 20^th^ century when compared to its values during most part of the 19^th^ century with a steep trend post 1970. The 2 ‰ range of variability in core sediments is similar to the spatial variability of δ^13^C of surface sediments along a transect between the inner fjord and the outer fjord. The δ^15^N of sediment core varied between 4.93 ‰ and 4.22 ‰ with a mean of 4.57 ‰ (Fig 4E). Trends are similar to those shown by TOC and δ^13^C during the 19^th^ century, however, its correspondence with TOC and δ^13^C is significantly suppressed during most part of the 20^th^ century. The most enriched values were observed at AD 1900 and AD 1840, concurrent with maxima in TOC and minima in δ^13^C. δ^15^N is in general lower during the 20^th^ century in comparison to the 19^th^ century. Overall, the range of variability in core sediments is less than its axis wide spatial variability [39] in surface sediments between the glacially influenced inner fjord and the open outer fjord.

### Foraminifera microfossil assemblage in surface and core sediments

**Fig 5:**
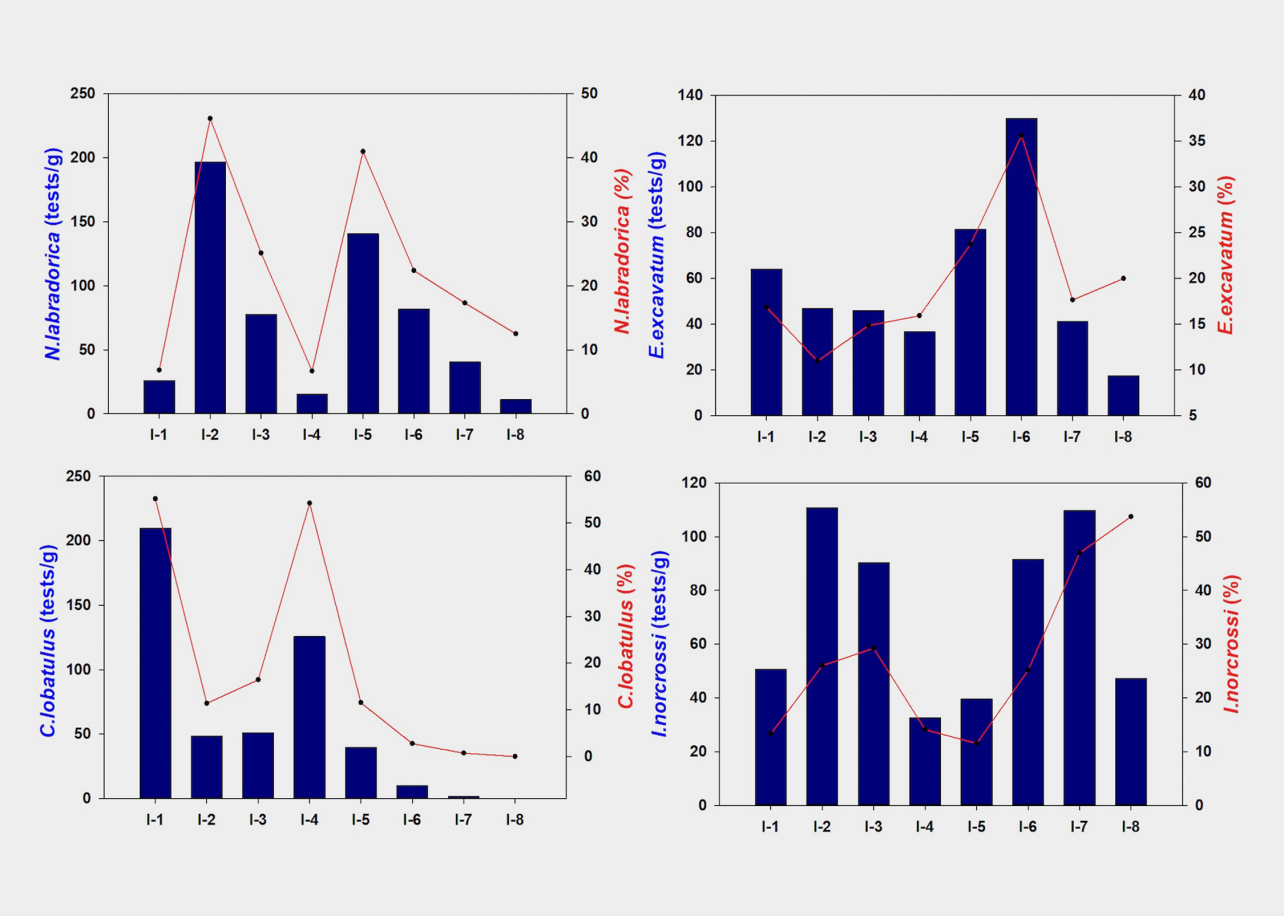
**Relative (continuous line) and absolute (solid bars) abundance of (a) *Nonionellena labradorica*, *(b) Elphidium excavatum*, (c) *Cibicides lobatulus and (d) Islandiella norcrossi* in surface sediments at Kongsfjorden**.

Benthic foraminifera tests constituted the bulk of the microfossil abundance as the planktic species were found to be very low in the surface and core sediments at Kongsfjorden. Dominant species constituting between 40% and 80% of the total tests in surface sediments included *Nonionellena labradorica*, *Elphidium excavatum*, *Islandiella norcrossi* and *Cibicides lobatulus* (Fig 5). Other common species found in the sediments with relatively low abundance included *Quinquelloculina stalkeri*, *Buccella frigida*, and *Cassudilina reniforme*. Along the fjord axis, the total foraminifera abundance (tests/g) varied from around 400 near the fjord mouth to less than 100 at the head. *Nonionellena labradorica*, known for its association with productivity as it feeds on phytodetritus [43,44], is found to be the most abundant species in surface sediments (22% relative abundance) and second most dominant species after *Islandiella norcrossi* (26%) in core sediments. An increasing trend in its abundance between the fjord head and the fjord mouth seems to originate from an increase in marine productivity along the fjord axis. The trend is however punctuated with drastically lower values at station I-4, a sill (water depth 180 m) and outermost station I-1, which lies close to frontal disturbances. These two stations further record a very high abundance of *Cibicides lobatulus*, which is known for its association with high current regimes [45,46] suggesting high water currents associated with shallow water at I-4 and proximity to frontal eddies at the outermost station I-1 as the reason for such a distribution. *Islandiella norcrossi* and *Elphidium excavatum* (27% and 20% relative abundance, respectively) showed a decreasing trend between the fjord head and the fjord mouth in terms of relative abundance. No such trend however in terms of absolute composition (tests/g) is present in case of *Islandiella norcrossi*. *Elphidium excavatum*, an opportunistic species [47,48] is found in high abundance in the inner fjord region.

Like the surface sediments, the assemblage represented by *N*. *labradorica*, *Elphidium excavatum*, and *Islandiella Norcrossi* is found to be dominant in core sediments too. *Islandiella Norcrossi* is found to be the most dominant benthic species with a mean composition of 311 tests/g (31.9% relative abundance) followed by *Nonionellena labradorica* and *Elphidium excavatum* with a mean composition of 266 tests/g (27% relative abundance) and 238 tests/g (24.2% relative abundance) respectively. While, the absolute abundance of *Nonionellena labradorica* showed an overall decreasing trend between AD 1810 and AD 2010, synchronous peaks with respect to geochemical proxies are observed for both its absolute and relative abundance (Fig 4F and 4G).

## Discussion

Modern observation based on water column measurements suggests the existence of steep productivity gradient along the axis of the fjord at Kongsfjorden. Lower productivity estimates in the inner fjord compared to the outer part are widely reported in various studies at Kongsfjorden [15,28]. Patterns of glacial sedimentation and its impact on the depth of the euphotic zone [31,32,33] point towards a link between glacial melting and primary production at Kongsfjorden. Geochemical parameters measured in surface sediments captures the axis wide gradient in productivity between the inner and the outer fjord. Thus, productivity proxies in core sediments can provide a reliable history of past glacial activity if marine encroachment from the other side does not significantly impact the records during the interval.

### Age model

Linking local productivity variability at our site with large scale regional changes requires a valid chronology of sediment core samples. Here we have used a combination of two different techniques (radioisotope dating and use of tie-points) to establish the overall chronology. Excess ^210^Pb measurements are widely used to estimate sedimentation rates in young sediments from lakes and shallow marine environments. However, establishing sediment ages based entirely on ^210^Pb activity requires a thorough exercise in quantitative process modeling due to various complications [49]. The upper few centimeters of the ^210^Pb depth profiles are particularly prone to complications due to both physical (bioturbation) and chemical (mobilization) factors. Whereas physical mixing would produce a homogenous profile of ^210^Pb in the top layer, studies have also shown post-depositional mobility of ^210^Pb due to diffusion [50]. We could not rule out these two factors at our site as visual inspection of ^210^Pb depth profile showed somewhat mixed values in the top few centimeters (S1 Fig). However, below this disturbed zone, the profile showed a smooth log-linear decline with high regression coefficient (r^2^ = 0.94), testifying the validity of the CIC model in the top 11 cm of the core. Below this depth, tie-points were selected based on peak excursion in multiple proxies. Since the use of tie-points induces some degree of circularity, it becomes important to examine the robustness of the age model thus generated. In the age model used in this study, the first tie-point is independently supported by ^210^Pb dating. With a sedimentation rate of 1 mm/y obtained from ^210^Pb profile, the first tie-point at 11.5 cm depth also yields an approximate age of 115 years which overlaps reasonably well with the first tied age. The criteria used for selecting the first tie-point (peak excursions in multiple proxies) was also applied to the remaining two tie-points. Since, the first tie-point is independently validated, the criteria itself gets validated to a reasonable extent and justifies the selection of the remaining tie-points. Further, the order of sedimentation rates obtained using ^210^Pb dating and those shown by the tie-points are similar. Apart from the points of peak excursions in the records, a substantial part of the discussion of proxy data is based on comparing trends between proxies and the temperature. If the tie-points were not reasonably accurate, the correspondence between the trends in the temperature record and the proxies would not have been possible. For instance, the warming trend shown by the first band in Fig 4 and between the third and the second band in Fig 4 matches well with the proxy records. Reasonably good match between the trends is an independent outcome of the age model as it was not part of the criteria applied for selecting the tie-points. Therefore, except for the two peak events (2^nd^ and 3^rd^ tie-points), for large part of the record, the argument of circularity does not seem to apply and even for the two tie-points, the selection criteria is fairly supported by ^210^Pb dating.

### Geochemical and microfossil proxies of productivity and their link to temperature

Large amplitude temporal variabilities, comparable to the axis-wide spatial variabilities as seen in surface sediments, was observed in a suite of geochemical proxies from the core sediments during the last two centuries. TOC data alone can serve as a good proxy for productivity and by extension for glacial melting at Kongsfjorden. Even if there are contribution due to clastic dilution, it can still be traced to glacial melting at this site as melt-water is known to increase material input into the fjord. Thus, TOC record of the core seems to reflect changes in glacial melting primarily on account of productivity changes due to increase in turbidity and possibly with contribution due to its direct dilution with melt driven changes in inorganic input. An advantage of reporting δ^13^C variability alongside TOC data is that it is not affected by clastic dilution if such a change does not drastically alter the terrestrial organic carbon supply. δ^13^C of marine organic matter at Kongsfjorden is reported to be lower than the δ^13^C of the terrestrial carbon supplied at Kongsfjorden via runoff [39]. Therefore, low δ^13^C values in core sediments suggest periods with high marine production whereas high values correspond to periods with lower in-situ production. We found a moderately strong negative correlation between TOC and δ^13^C in our record which further suggests the applicability of δ^13^C as a tracer for productivity and most likely for changing glacial activity at Kongsfjorden. Lack of an even higher correlation between TOC and δ^13^C seems to result from the role of clastic dilution on TOC record as δ^13^C would be relatively less sensitive to such dilution. Productivity changes can further be recorded by δ^15^N if the relation is not complicated by other factors. We observed that while there was a good correspondence between δ^15^N and other productivity proxies during the 19^th^ century, the same appeared subdued during parts of the 20^th^ century suggesting the presence of other controls in addition to productivity. For example, positive correlation between productivity and δ^15^N under normal conditions will be suppressed in presence of a strong stratification of the water column. In such a case, even during intervals with low productivity, δ^15^N may not decline or in extreme cases may even show an increase due to lower availability of nitrate in the top stratified layer.

Proxy records from the sediment core are compared with the instrumental temperature record (10-year smoothed mean annual air temperature record from Svalbard airport [51]; available only for the 20^th^ century) and Austfonna ice-core record from Svalbard (Fig 4A and 4B). During the overlapping period, instrumental and ice-core record shows good correspondence, suggesting the applicability of Austfonna ice-core record as a reliable proxy for temperature during the 19^th^ century for which direct measurements are not available. All the three geochemical productivity proxies showed a gradual decline in productivity during large part of the 19^th^ century except for a sharp negative swing during the 1820s (4^th^ band in Fig 4) and a short-lived positive swing centered around AD 1840 (3^rd^ band in Fig 4). Both Short lived excursions and the general declining trend during the 19^th^ century appears to be driven by temperature when compared with the ice-core record. Geochemical proxies suggest a sharp increase in productivity at the turn of the century around AD 1900 (2^nd^ band in Fig 4) which again appears to be temperature driven. Peak minimum temperatures are seen through the instrumental and the ice core record during this period. Thereafter, proxy data shows a declining trend in productivity which is more pronounced after 1970 (1^st^ band in Fig 4). Instrumental observations show strong positive decadal trends in temperature post late 1960s suggesting temperature driven glacial melting as a dominant control on the productivity trend during the period. Interestingly, despite a common general trend in all the three geochemical proxies throughout the span of the core, the correlation coefficient between δ^15^N and other productivity proxies (TOC and δ^13^C) was low during the 20^th^ century.[52] observed that with warming the advection of the Atlantic water into the fjord occupies a much shallower depth compared to the time when the AW core is colder which causes stratification in the upper layers. It appears that the weakening of correlation between δ^15^N and TOC/δ^13^C during the 20^th^ century is possibly due to an increase in water column stratification with warming.

Past productivity changes at Kongsfjorden, reconstructed based on the geochemical and the stable isotopic record of sediments is further supported by changes in benthic microfossil assemblage over the period. Surface foraminifera assemblage shows a clear zonation largely as a result of the glacial marine contrast at the fjord. Similar to the geochemical and the isotopic distribution at Kongsfjorden, a marked increase in test concentration, as well as species diversity, is seen along the increasing salinity gradient from fjord head to open ocean which suggests foraminifera assemblage data of core sediments may reflect changes in glacial activity in the past. Evidence of a decline in productivity during the last two centuries can be seen through a fall in the relative and absolute abundance of *N*. *labradorica* in core sediments, a benthic species known for its association with productivity.

## Conclusion

Glacial melting at Kongsfjorden may have its source in regional scale changes, particularly the warming at high latitudes. Linking productivity with glacial melting allows us a qualitative reconstruction of past melting history at Kongsfjorden based on productivity changes. Multiple proxies are used to reconstruct these changes during the last two centuries. The interpretation of results from the core sediments is further validated by an extensive study of the modern dynamics based on the analysis of the surface sediments distributed along the fjord axis. Results show productivity variability captures both the general warming trend as well as abrupt changes in temperature during the last two centuries.

## Supporting information

S1 Fig Depth profiles of ^226^Ra and ^210^Pb. (TIFF)

S2 Fig TOC and δ^13^C relation during 19^th^ and 20^th^ century. (TIF)

S1 Supplementary Information Surface sediment and core sediment data. (PDF)
