# Supplementary figures and images for: Warming in the Arctic Captured by productivity variability at an Arctic Fjord over the past two centuries
Source: PLoS One. 2018 Aug 15;13(8):e0201456. doi: 10.1371/journal.pone.0201456 (PMC6093672; doi:10.1371/journal.pone.0201456)

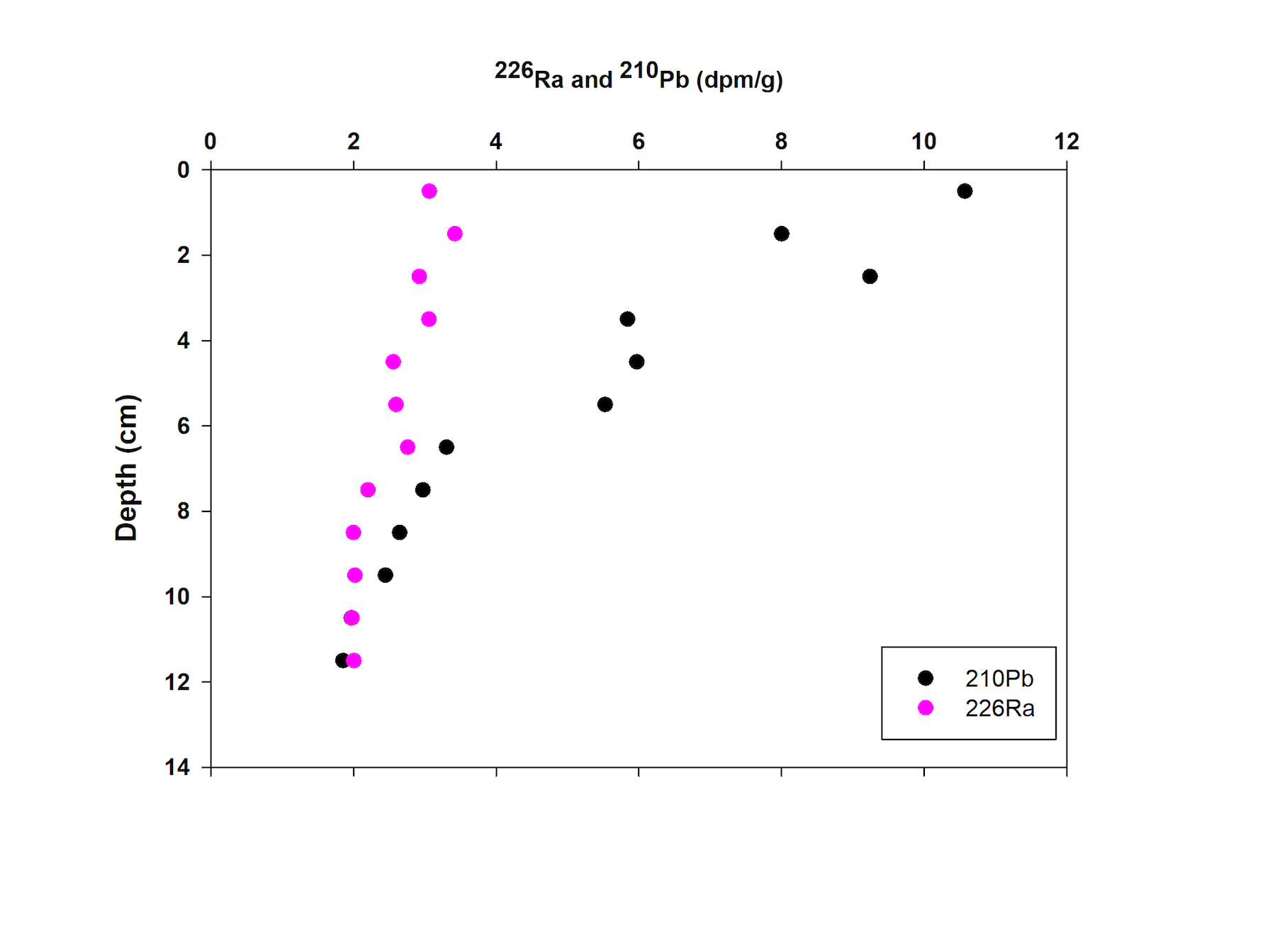

Supplement: S1 Fig — (TIFF) [file pone.0201456.s001.tiff]

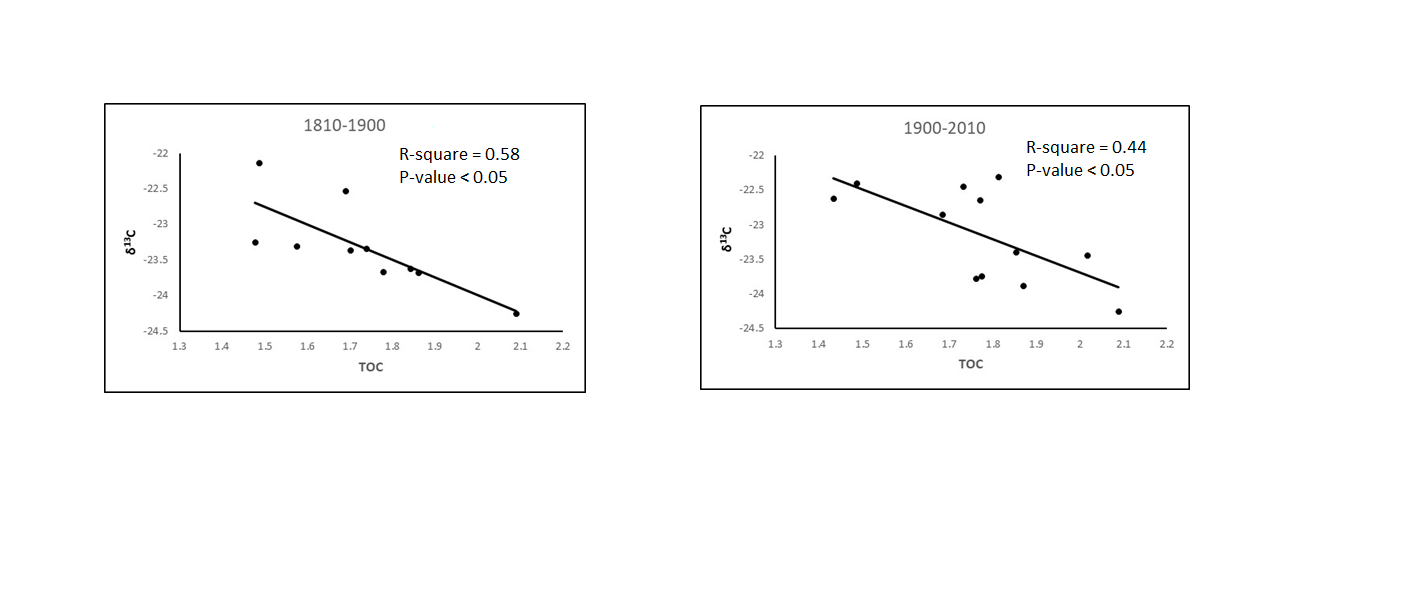

Supplement: S2 Fig — (TIF) [file pone.0201456.s002.tif]
