# Supplementary material for: Warming in the Arctic Captured by productivity variability at an Arctic Fjord over the past two centuries
Source: PLoS One. 2018 Aug 15;13(8):e0201456. doi: 10.1371/journal.pone.0201456 (PMC6093672; doi:10.1371/journal.pone.0201456)
Supplement: S1 Supplementary Information — (PDF) [file pone.0201456.s003.pdf]

Supplementary Information

Warming in the Arctic Captured by Productivity Variability at an Arctic Fjord over the Past Two Centuries

Vikash Kumar[1], Manish Tiwari[1]* & R. Rengarajan[2]

1. National Centre for Antarctic & Ocean Research, Vasco-da-Gama, Goa-403804, India

2. Physical Research Laboratory, Navrangpura, Ahmedabad-380009, India

* Corresponding Author: manish@ncaor.gov.in

Table A. Core sediment data

Depth (cm)	Age	TOC(%)	$\delta^{13}C$	$\delta^{15}N$	N.labradorica (tests/g)	N.labradorica (%)	^{210}Pb (dpm/g)	^{226}Ra (dpm/g)
0.50	2010	1.77	-22.64	4.72	77.78	25.13	10.57	3.06
1.50	2000	1.43	-22.61	4.80	54.44	18.63	8.00	3.42
2.50	1990	1.49	-22.40	4.22	59.23	17.00	9.24	2.92
3.50	1980	1.68	-22.85	4.54	219.51	22.31	5.84	3.06
4.50	1970	1.76	-23.78	4.56	135.20	25.33	5.97	2.56
5.50	1960	1.73	-22.44	4.66	206.64	28.00	5.53	2.59
6.50	1950	1.87	-23.88	4.07	166.91	29.20	3.30	2.76
7.50	1940	1.77	-23.74	4.87	182.32	27.62	2.97	2.20
8.50	1930	1.85	-23.39	4.27	277.91	38.49	2.65	2.00
9.50	1920	2.02	-23.44	4.31	380.43	30.76	2.45	2.02
10.50	1910	1.81	-22.31	4.78	205.71	25.09	1.96	1.98
11.50	1900	2.09	-24.25	4.91	779.55	36.72	1.85	2.01
12.50	1885	1.48	-23.25	4.59	298.69	27.65		
13.50	1870	1.57	-23.31	4.59	379.49	22.09		
14.50	1855	1.78	-23.66	4.71	116.13	14.94		
15.50	1840	1.84	-23.62	4.93	527.59	29.25		
16.50	1834	1.74	-23.34	4.73	384.62	22.14		
17.50	1828	1.49	-22.13	4.71	216.22	26.25		
18.50	1822	1.70	-23.36	4.53	284.69	24.49		
19.50	1816	1.69	-22.53	4.30	341.67	32.37		
20.50	1810	1.86	-23.68	4.30	345.71	37.93		

Table B. Surface sediment data

Station	*N.labradorica* (tests/g)	*E.excavatum* (tests/g)	*I.norcrossi* (tests/g)	*C.lobatulus* (tests/g)
I-1	25.93	64.20	50.62	209.88
I-2	196.88	46.88	110.94	48.44
I-3	77.78	46.03	90.48	50.79
I-4	15.51	36.90	32.62	125.67
I-5	140.74	81.48	39.51	39.51
I-6	81.67	130.00	91.67	10.00
I-7	40.50	41.32	109.92	1.65
I-8	10.99	17.58	47.25	0.00
